# Association of *Staphylococcus aureus* Colonization and Pneumonia in the Intensive Care Unit

**DOI:** 10.1001/jamanetworkopen.2020.12741

**Published:** 2020-09-30

**Authors:** Fleur P. Paling, Derek Hazard, Marc J. M. Bonten, Herman Goossens, Hasan S. Jafri, Surbhi Malhotra-Kumar, Frangiscos Sifakis, Susanne Weber, Jan A. J. W. Kluytmans

**Affiliations:** 1 Julius Center for Health Sciences and Primary Care, University Medical Center Utrecht, Utrecht University, Utrecht, the Netherlands; 2 Institute of Medical Biometry and Statistics, University Medical Center Freiburg, Freiburg, Germany; 3 Department of Medical Microbiology, University Medical Center Utrecht, Utrecht University, Utrecht, the Netherlands; 4 Laboratory of Medical Microbiology, Vaccine and Infectious Disease Institute, University of Antwerp, Antwerp, Belgium; 5 Microbial Sciences, R&D BioPharmaceuticals, AstraZeneca, Gaithersburg, Maryland; 6 Boehringer Ingelheim Pharmaceuticals Inc, Ridgefield, Connecticut; 7 Department of Infection Control, Amphia Hospital, Breda, the Netherlands

## Abstract

**Importance:**

Carriage of *Staphylococcus aureus* is associated with *S aureus* infection. However, associations between *S aureus* carriage and the development of *S aureus* intensive care unit (ICU) pneumonia (SAIP) have not been quantified accurately, and interpretation of available data is hampered because of variations in definitions.

**Objective:**

To quantify associations of patient-related and contextual factors, including *S aureus* colonization status, with the occurrence of SAIP.

**Design, Setting, and Participants:**

This cohort study was conducted in ICUs of 30 hospitals in 11 European countries, geographically spread across 4 regions. Among patients with an anticipated length of stay 48 hours or longer who were undergoing mechanical ventilation at ICU admission, *S aureus* colonization was ascertained in the nose and lower respiratory tract. From this group, *S aureus*–colonized and noncolonized patients were enrolled into the study cohort in a 1:1 ratio. Data analysis was performed from May to November 2019.

**Main Outcomes and Measures:**

SAIP was defined as any pneumonia during the ICU stay developing 48 hours or more after ICU admission with *S aureus* isolated from lower respiratory tract specimens or blood samples. The incidence of SAIP was derived in the study cohort and estimated on the weighted incidence calculation for the originating overarching population, while taking competing events into account. Weighted risk factor analysis was performed using Cox multivariable regression.

**Results:**

The study cohort consisted of 1933 patients (mean [SD] age, 62.0 [16.0] years); 1252 patients (64.8%) were men, and 950 patients (49.1%) were *S aureus* carriers at ICU admission. In all, 304 patients (15.7%) developed ICU-acquired pneumonia, of whom 131 patients (6.8%) had SAIP. Weighted SAIP incidences were 11.7 events per 1000 patient-days in the ICU for *S aureus–*colonized patients and 2.9 events per 1000 patient-days in the ICU for noncolonized patients (overall incidence, 4.9 events per 1000 patient-days in the ICU). The only factor independently associated with SAIP was *S aureus* colonization status at ICU admission (cause-specific hazard ratio, 3.6; 95% CI, 2.2-6.0; *P* < .001). There were marked regional differences in SAIP incidence and cause-specific hazard ratios for colonization status.

**Conclusions and Relevance:**

SAIP incidence was 4.9 events per 1000 ICU patient-days for patients undergoing mechanical ventilation at ICU admission (or shortly thereafter). The daily risk of SAIP was 3.6 times higher in patients colonized with *S aureus* at ICU admission compared with noncolonized patients.

## Key Points

**Question:**

What is the incidence density of *Staphylococcus aureus* intensive care unit pneumonia (SAIP) in Europe, and which factors are associated with the risk of SAIP?

**Findings:**

In this cohort study of 1933 participants, the weighted incidence density of SAIP was 4.9 events per 1000 intensive care unit patient-days, and *S aureus* colonization was the only factor independently associated with SAIP.

**Meaning:**

These findings suggest that SAIP incidence may be higher than initially perceived, and future interventions to prevent SAIP should focus on patients colonized with *S aureus* to achieve a higher efficacy.

## Teaser

This cohort study analyzes the association of *Staphylococcus aureus* colonization status with the occurrence of *S aureus *pneumonia among European patients in the intensive care unit.

## Introduction

*Staphylococcus aureus* is both a human commensal and an opportunistic pathogen. Healthy people carry the bacterium on the skin or in the respiratory tract, with a preference for the nose. Reported rates of nasal carriage are approximately 25% to 30% of healthy individuals.^1,2^ For healthy people, *S aureus* carriage is not a direct risk for infection,^3^ but this changes in case of surgery or serious illness, such as when being treated in an intensive care unit (ICU). Although *S aureus* infections do occur in noncarriers, they occur far more frequently in those who are colonized with *S aureus*.^4,5^ Nosocomial pneumonia caused by *S aureus* frequently complicates hospitalization and may lead to severe consequences, especially when acquired in the ICU.^6,7^

However, little is known about the incidence of *S aureus* ICU pneumonia (SAIP) and about variations in incidence between and within countries, which partly results from differences in definitions and diagnostic detection methods used in previous studies.^5,8^ Furthermore, for SAIP specifically, risk factors have not been quantified adequately. Apart from colonization status, other patient-related factors could increase the risk of developing SAIP. The ASPIRE-ICU (Advanced Understanding of *Staphylococcus aureus* and *Pseudomonas aeruginosa* Infections in Europe–ICU) study^9^ was designed to quantify associations between patient-related and contextual factors, including *S aureus* colonization status at the time of ICU admission and the occurrence of SAIP in 11 European countries.

## Methods

### Study Design, Setting, and Participants

ASPIRE-ICU was a cohort study of adult ICU patients at 30 hospitals in 11 European countries that recruited participants between June 2015 and October 2018. The study rationale and methods have been reported elsewhere.^9^ The study protocol was approved by the institutional review boards or ethical review committees in each country and/or site. This study was conducted according to the principles of the Declaration of Helsinki, in accordance with the Medical Research Involving Human Subjects Act and local guidelines in the participating countries. This study follows the Strengthening the Reporting of Observational Studies in Epidemiology (STROBE) reporting guideline.

For this study, we identified ICUs with routine admission screening for *S aureus* carriage in the nose and lower respiratory tract (LRT) in patients with an expected length of stay (LOS) of 48 hours or more and who underwent mechanical ventilation at ICU admission (or were expected to undergo ventilation within 24 hours). In summary, the study considered 2 populations: an overarching source population consisting of consecutive patients admitted to the ICU with an expected LOS of 48 hours or more and undergoing mechanical ventilation at ICU admission (or expected to undergo ventilation within 24 hours), and the study cohort consisting of participants from the source population who provided written, informed consent for additional data and sample collection. Primary outcomes were derived from the study cohort. The source population was used to derive weighted incidence estimates using basic surveillance data and to determine differences between patients enrolled and not enrolled in the study cohort.

Participants from whom both a nose and LRT screening sample could be obtained at ICU admission were eligible for the study cohort. LRT samples included endotracheal aspirate, spontaneously produced sputum, or throat swabs if aspirates and sputum were not available. We aimed to enroll 2000 study cohort participants within 3 days after ICU admission, in a 1:1 ratio of *S aureus*–colonized and noncolonized patients. We enrolled all *S aureus* carriers in each ICU and approached the first eligible noncarrier after each enrolled *S aureus* carrier. Other inclusion and exclusion criteria and sample size calculations are described elsewhere.^9^ Patients with *S aureus* pneumonia at ICU admission were excluded from this analysis. During ICU stay, study samples (eg, endotracheal aspirates) were obtained 3 times weekly during the first week, 2 times weekly during the 3 weeks thereafter, and during each day of protocol-defined pneumonia, as described elsewhere.^9^ Criteria for establishing SAIP diagnosis were evaluated daily, as were the results from diagnostic tests performed during ICU stay for clinical reasons.

In each region in Europe, as described by the United Nations, we included at least 1 country.^10^ A list of participating countries, including the final number of enrolled participants per country, can be found in eTable 1 in the Supplement.

### Study End Points

The primary outcome (incidence of SAIP through ICU stay) was assessed in several steps. First, the following 4 clinical criteria were assessed daily: any new antibiotic use, new blood cultures performed, new chest radiograph or computed tomography scan that shows a new or worsening infiltrate, or other new reason to suspect pneumonia. In cases with 1 positive answer, a combination of objective major and minor criteria was assessed to categorize patients as having protocol-defined pneumonia or not, as described elsewhere.^9^ The primary end point, SAIP, was determined post hoc on the basis of isolation of *S aureus* from any LRT specimen (including both clinical and study surveillance cultures) or blood culture in the 3 days before and after the day of pneumonia diagnosis. Secondary outcomes included all-cause ICU-acquired pneumonia and mortality at days 30 and 90 after ICU admission.

### Laboratory Methods

*S aureus* screening samples were processed locally on chromagar plates (Colorex staph aureus; Biotrading) using standardized methods. *S aureus* strains were selected on phenotypic criteria (pink or mauve color) and shipped to the central study laboratory. All predefined study samples were frozen at −80 °C and also shipped to the central laboratory. *S aureus* isolates from screening and clinical samples from patients with SAIP were compared using multilocus sequence typing.

### Statistical Analysis

#### Incidence Calculation and Primary End Points

The incidence of SAIP was determined in the study cohort and estimated for the source population using weighting methods. The weighting methods used the observed proportion of *S aureus* carriage in the source population in combination with the likelihood of patients to be included as study participants, stratified per country to calculate the incidence density estimate for the overall source population. These methods are described in more detail in the eAppendix in the Supplement. Unweighted incidence calculations for the study participants also are provided in the eAppendix in the Supplement. Incidence density is described by *S aureus* colonization status and region using a Cox survival analysis and taking into account the competing events death and ICU discharge without SAIP.^11,12^ Cumulative incidence curves were plotted.^13,14^

#### Risk Factor Analysis

Cause-specific hazards were determined for SAIP and the competing events, representing the daily risk for a patient at a specific time to acquire each event. The next step was a weighted risk factor analysis for each competing event, yielding univariable cause-specific hazard ratios (CSHRs) per exposure status. Because of anticipated differences between countries, the cause-specific Cox model was stratified per country. Finally, a multivariable Cox regression survival analysis was performed, using variables selected from the univariable analysis to quantify the cumulative risk of acquiring SAIP in the presence of competing events. Two-sided *P* < .05 was considered significant. Statistical analyses were performed using R statistical software version 3.6.1 (R Project for Statistical Computing).^15^ Data analysis was performed from May to November 2019.

#### Variable Selection

For univariable analysis, the following variables were selected before analysis, on the basis of clinical reasoning and published data: *S aureus* colonization status, sex, body mass index (calculated as weight in kilograms divided by height in meters squared), APACHE (acute physiology, age, chronic health evaluation) IV score, origin before ICU stay, prior antibiotic use (defined as any systemic antibiotic use for ≥1 day within the 2 weeks before ICU stay), neurotrauma (admitted for trauma and Glasgow Coma Scale score of ≤8), pneumonia diagnosis, active *S aureus* infection (other than pneumonia), diabetes, bed head elevation during ICU stay, and peptic ulcer prophylaxis during ICU stay. Unless stated otherwise, all variables were measured at ICU admission. Variables such as age, chronic pulmonary disease, immunodeficiency status, and mechanical ventilation were not included because of overlap with the APACHE IV score and/or inclusion criteria. Age, APACHE IV score, and body mass index were included as continuous variables. Variables were selected for the multivariable model in case they yielded *P* < .157 (roughly corresponding to the Akaike information criterion) in any of the competing events’ univariable analysis, abiding by the rule of 1 covariate per 10 events.^16,17^ Missing data on risk factors were imputed using multiple imputation methods.

#### Sensitivity Analysis

To determine the robustness of results we performed several sensitivity analyses all on unweighted data. First, we checked whether exclusion of 26 patients from 1 site changed results, because contact with the site was lost at the end of participants recruitment and data could not be verified. Second, we determined to what extent excluding patients with missing pneumonia information on at least 2 days and for 30% or more of days in total influenced results. Finally, the complete analysis was repeated 11 times, each time excluding 1 country.

## Results

In all, 1933 participants were included in the study cohort; 950 patients (49.1%) were colonized with *S aureus* at ICU admission. The mean (SD) age was 62.0 (16.0) years, and 1252 patients (64.8%) were men (Table 1).

**Table 1.** Baseline Characteristics of Patients

Characteristic	Patients, No. (%)		
	*S aureus* positive	*S aureus* negative	Total
Age, mean (SD), y	60.8 (17.1)	63.1 (14.8)	62.0 (16.0)
Sex			
Male	634 (66.7)	618 (62.9)	1252 (64.8)
Female	316 (33.3)	365 (37.1)	6801 (35.2)
Origin before ICU stay^a^			
Home or community	567 (59.7)	491 (49.9)	1058 (54.7)
Health care related	382 (40.2)	488 (49.6)	870 (45.0)
APACHE IV score, mean (SD)^b^	72.2 (38.2)	72.0 (37.9)	72.1 (38.0)
Body mass index, mean (SD)^c^	27.2 (6.4)	27.4 (5.9)	27.3 (6.1)
Region			
North	123 (12.9)	128 (13.0)	251 (13.0)
South	411 (43.3)	411 (41.8)	822 (42.5)
East	193 (20.3)	202 (20.5)	395 (20.4)
West	223 (23.5)	242 (24.6)	465 (24.1)
Admission specialty			
Medical	484 (50.9)	464 (47.2)	948 (49.0)
Trauma	204 (21.5)	169 (17.2)	373 (19.3)
Surgical cardiothoracic	49 (5.2)	74 (7.5)	123 (6.4)
Surgery^d^			
Surgical other	213 (22.4)	276 (28.1)	489 (25.3)
Emergency	286 (30.1)	338 (34.4)	624 (32.3)
Elective	76 (8.0)	98 (10.0)	174 (9.0)
Nonsurgical	588 (61.9)	547 (55.6)	1135 (58.7)
Neurotrauma^b^			
Yes	120 (12.6)	86 (8.7)	206 (10.7)
No	830 (87.4)	897 (91.3)	1727 (89.3)
Prior antibiotic use			
Yes	179 (18.8)	293 (29.8)	472 (24.4)
No	674 (70.9)	600 (61.0)	1274 (65.9)
Unknown	97 (10.2)	92 (9.2)	187 (9.7)
Diabetes^e^			
Yes	183 (19.3)	199 (20.3)	382 (19.8)
No	766 (80.7)	783 (79.7)	1549 (80.1)
Pneumonia^b^^,^^f^			
Yes	142 (14.9)	184 (18.7)	326 (16.9)
No	807 (84.9)	797 (81.1)	1604 (83.0)
Active *S aureus* infection^b^^,^^g^^,^^h^			
Yes	39 (4.1)	15 (1.5)	54 (2.8)
No	910 (95.8)	965 (98.2)	1875 (97.0)
Total	950 (100)	983 (100)	1933 (100)

Abbreviations: APACHE, Acute Physiology, Age, Chronic Health Evaluation; ICU, intensive care unit.

^a^ Status was unknown for 5 patients.

^b^ Refers to status at ICU admission.

^c^ Body mass index is calculated as weight in kilograms divided by height in meters squared.

^d^ In case a trauma patient needed surgery related to this trauma, this was assumed to be emergency surgery.

^e^ Data were missing for 2 patients.

^f^ Status was unknown for 3 patients.

^g^ Refers to infections other than pneumonia at ICU admission.

^h^ Status was unknown for 4 patients.

In the source population, 9841 patients were included. Of these, 2440 patients (24.8%) had *S aureus* colonization, 6838 (69.5%) had negative screening results, and colonization status could not be determined for 563 patients (5.7%). In 445 patients (4.5%), either a nasal or LRT sample was missing and *S aureus* colonization status was based on 1 available sample. Seventy patients were classified as *S aureus* carriers and 375 were classified as noncarriers.

Most baseline characteristics were comparable between the source population and the study cohort, as were ICU mortality rates (see eTable 2 in the Supplement). Study cohort participants had slightly longer ICU stay (mean difference, 1.3 days).

### Patient Flow

**Figure 1.**
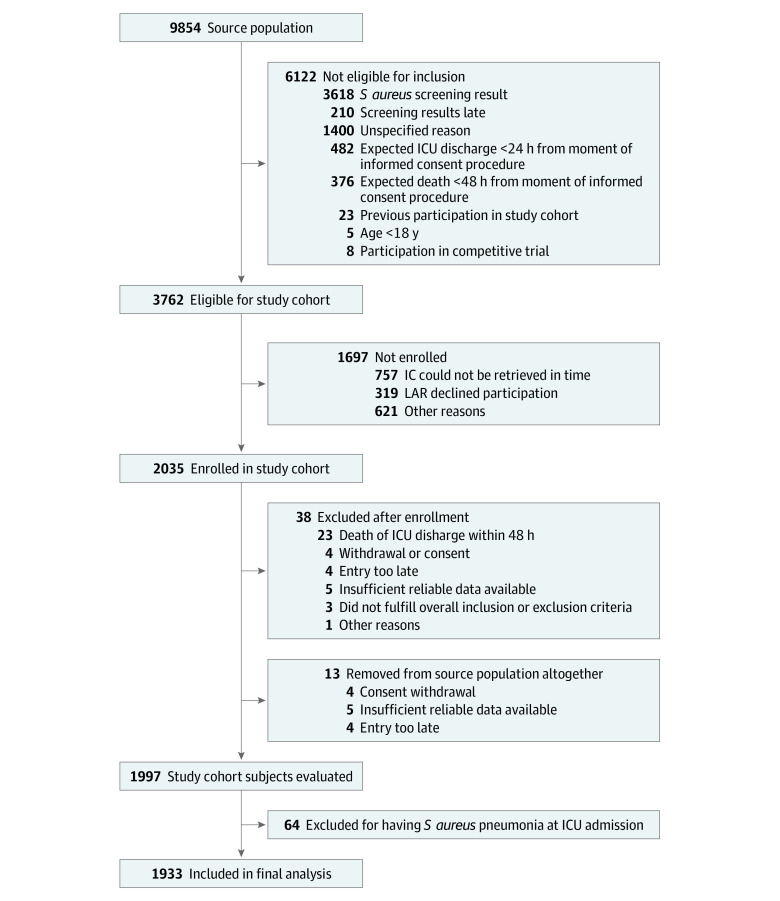
Flowchart of Patients With *Staphylococcus aureus* Pneumonia The final evaluable number of patients in the source population is 9841, because of the 13 participants who were removed after enrollment in the study cohort. IC, indicates informed consent; ICU, intensive care unit; LAR, legally accepted representative.

In all, 9854 patients were screened, of whom 6122 were considered ineligible for participation in the study cohort (Figure 1). Of the 3732 patients considered eligible, 2035 provided informed consent and were enrolled in the study cohort. Thirty-eight patients were nonevaluable, in 23 cases because of an LOS of less than 48 hours. For the current analysis, 64 patients with *S aureus* pneumonia at ICU admission were excluded, resulting in a study cohort of 1933 participants for this analysis.

### Incidence of SAIP

**Figure 2.**
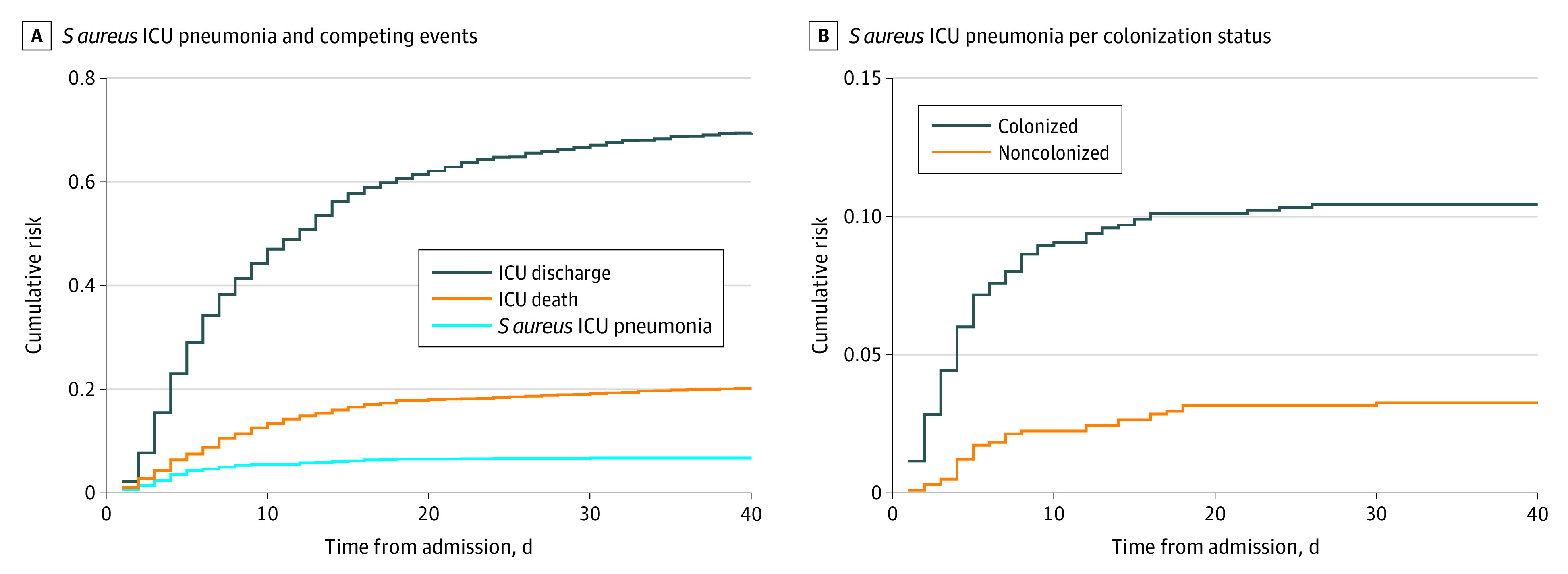
Cumulative Incidence Functions for *Staphylococcus aureus* Pneumonia Among Patients in the Intensive Care Unit (ICU) Panel A shows incidence rates for *S aureus* ICU pneumonia and its competing events. Panel B shows incidence rates for *S aureus* ICU pneumonia per colonization status.

ICU-acquired pneumonia was observed in 304 patients (15.7%), 131 of whom (6.8%) were categorized as having SAIP, on the basis of either local (74 patients) and/or central laboratory (120 patients) culture results (see eTable 3 in the Supplement). The weighted incidence estimate for SAIP in the original source population was 4.9 events per 1000 days at risk. Weighted incidences were 11.7 events per 1000 days at risk for *S aureus*–colonized patients and 2.9 events per 1000 days at risk for noncolonized patients. SAIP incidences differed between regions, as did associations between *S aureus* carriage and the occurrence of SAIP (Table 2). The incidence of SAIP in *S aureus* carriers ranged from 17.6 events per 1000 days in the northern region to 6.2 events per 1000 days in the southern region. The median time to SAIP varied from 3.0 days for colonized patients in the western region to 7.5 days in noncolonized patients in the southern region. Weighted cumulative incidence functions for SAIP per colonization status demonstrate that in *S aureus* carriers, most SAIP episodes occurred during the first week of ICU admission (Figure 2A). The occurrence of SAIP in association with the competing events (ICU discharge and death) is depicted in Figure 2B. Unweighted incidence data, incidence numbers stratified for sample type *S aureus* positivity, and weighted cumulative incidence functions per region are provided in eTable 4, eTable 5, eTable 6, eTable 7, eFigure 1, eFigure 2, eFigure 3, eFigure 4, eFigure 5, and eFigure 6 in the Supplement. The mean number of microbiological cultures from respiratory samples and blood per participant that were locally obtained for clinical reasons varied between 0.29 to 0.74 per day over the different regions (eTable 8 in the Supplement).

**Table 2.** Weighted Incidence of SAIP

Variable	Patients, No.	Time at risk, d	Patients at risk, No. (%)	Rate, No. of patients/1000 d at risk	Time to SAIP, median, d
*S aureus *colonization status					
Positive	2204	22 266	261 (11.8)	11.7	4
Negative	7221	79 711	234 (3.2)	2.9	6
Region					
North	1894	22 161	162 (8.8)	7.3	5
South	2585	33 832	114 (6.3)	3.3	7
East	1057	11 090	56 (6.1)	5	5.5
West	3889	34 889	163 (7.1)	4.7	4
*S aureus* positive, region					
North	411	4152	73 (17.8)	17.6	4
South	709	9012	56 (7.9)	6.2	6
East	291	2858	33 (11.3)	11.5	5
West	793	6244	99 (12.5)	15.9	3
*S aureus* negative, region					
North	1483	18 009	89 (6.0)	4.9	6
South	1876	24 820	58 (3.1)	2.3	7.5
East	766	8232	23 (3.0)	2.8	6
West	3096	28 650	64 (2.1)	2.2	4
Overall	9425	101 977	495 (5.3)	4.9	5

Abbreviation: SAIP, *Staphylococcus aureus* ICU pneumonia.

### Colonizing vs Infecting Strains

Ninety-nine patients developed SAIP after prior *S aureus* colonization at ICU admission. Genetic comparison of *S aureus* isolates associated with colonization and infection within these individual patients was possible for 84 of 99 episodes, because of unavailability of either the infecting strain (10 episodes) or the colonizing strain (5 episodes) in the central laboratory. In 57 of these 84 paired strains (68%), multilocus sequence types were identical for the colonizing and infecting strains. Proportions of similarity ranged from 95% (19 of 20 pairs) in the western region to 49% (16 of 33 pairs) in the southern region. The most dominant multilocus sequence types were ST239 (19 strains, of which 11 were in 1 region) for infecting and ST30 (11 strains) for colonizing strains.

### Risk Factor Analysis

The univariable CSHR for developing SAIP for *S aureus*–colonized compared with noncolonized patients was 4.1 (95% CI, 2.5-6.9; *P* < .001). Pneumonia diagnosis at ICU admission (excluding those caused by *S aureus*) appeared to be protective against developing SAIP (CSHR, 0.4; 95% CI, 0.2-0.9; *P* = .03) (Table 3). CSHRs for death and ICU discharge without SAIP can be found in eTable 9 in the Supplement. On the basis of the univariable analysis, 11 variables were included in the multivariable analysis, yielding a CSHR of 3.6 (95% CI, 2.2-6.0; *P* < .001) to develop SAIP for colonized patients compared with noncolonized patients (Table 3). Unweighted CSHRs (including for competing events) are shown in eTable 10 and eTable 11 in the Supplement.

**Table 3.** Risk Factor Analysis for *Staphylococcus aureus* ICU Pneumonia

Risk factor	Univariable analysis		Multivariable analysis	
	CSHR (95% CI)	*P* value	CSHR (95% CI)	*P* value
*S aureus* colonization status^a^				
Colonized	4.12 (2.48-6.85)	<.001^b^	3.61 (2.17-6.03)	<.001^c^
Noncolonized	1 [Reference]		1 [Reference]	
Sex				
Male	0.89 (0.51-1.56)	.69	Not included	Not applicable
Female	1 [Reference]		1 [Reference]	
Origin before ICU stay				
Health care setting	0.73 (0.42-1.28)	.27	0.94 (0.44-2.00)	.87
Community origin before ICU stay	1 [Reference]		1 [Reference]	
APACHE IV score^a^^,^^d^	1.00 (0.99-1.00)	.52	1.01 (1.00-1.01)	.24
Body mass index^d^^,^^e^	0.96 (0.93-1.00)	.06^b^	0.97 (0.93-1.01)	.10
Neurotrauma^a^	1.89 (1.00-3.53)	.05^b^	1.23 (0.65-2.30)	.53
Prior antibiotic use	0.51 (0.23-1.12)	.09^b^	0.76 (0.27-2.13)	.60
Diabetes	0.93 (0.47-1.83)	.83	1.14 (0.56-2.32)	.73
Pneumonia^a^	0.44 (0.20-0.94)	.03^b^	0.53 (0.23-1.22)	.14
Active *S aureus* infection other than pneumonia^a^	2.18 (0.75-6.34)	.16^b^	1.51 (0.47-4.89)	.49
Peptic ulcer prophylaxis^f^	1.85 (0.66-5.17)	.24	1.72 (0.61-4.79)	.52
Bed head elevation^f^	0.66 (0.15-2.90)	.58	1.00 (0.22-4.66)	>.99

Abbreviations: APACHE, Acute Physiology, Age, Chronic Health Evaluation; CSHR, cause-specific hazard ratio; ICU, intensive care unit*.*

^a^ Refers to status at ICU admission.

^b^ Variables that univariably were associated with *P* < .157 (for *S aureus* ICU pneumonia or competing events) were included in final multivariable model.

^c^ Variable was significant in multivariable analysis.

^d^ Calculated per point increase.

^e^ Body mass index is calculated as weight in kilograms divided by height in meters squared.

^f^ Refers to events occurring during ICU stay.

### Sensitivity Analyses

Exclusion of the 26 patients with unverified data did not change results. There were 172 patients (8.9%) with missing pneumonia information on at least 2 days. For 79 of these patients (4.1% of the total), the amount exceeded 30% of the total amount expected; in 3 (3.8%) of these patients, SAIP was observed despite missing data. Exclusion of these patients did not change the interpretation. Exclusion of 1 specific country from the analysis changed the unweighted multivariable CSHR for *S aureus* colonization status from 3.1 (95% CI, 2.1-4.7) to 5.0 (95% CI, 3.0-8.5). Mean changes in CSHR after removing other individual countries were 0.1. No other clinically relevant of statistically significant estimate changes were observed in this sensitivity analysis.

## Discussion

In this international cohort study, patients colonized with *S aureus* at the time of ICU admission had an almost 4-fold higher risk of developing SAIP compared with noncolonized patients. Incidence densities for SAIP were 11.7 events per 1000 days at risk for *S aureus* carriers, 2.9 events per 1000 days at risk for noncarriers, and 4.9 events per 1000 days at risk for the total ICU population. The incidence of SAIP and the strength of the association between carriage and SAIP differed between European geographical regions, as did microbiological culture frequency. SAIP incidence was highest in the northern and lowest in southern Europe, and *S aureus* carriage had the largest risk for SAIP in western Europe.

To our knowledge, the observed regional differences in SAIP incidence and risks associated with *S aureus* colonization across Europe have not been reported earlier. Indeed, SAIP incidence may be associated with differences in diagnostic workup, which includes chest radiographs and microbiological cultures. The lowest SAIP incidence was observed in the region with the lowest culture frequency, and the second highest incidence was observed in the region with the highest culture frequency. Yet, a scatterplot of the associations between culture frequency and SAIP incidence per study site suggests that culture frequency alone cannot explain these associations (eFigure 7 in the Supplement). Unfortunately, the numbers of chest radiographs performed were not available.

Besides the differences in diagnostic strategies, regional differences in actual risk of SAIP associated with colonization status may also result from differences in sources and transmission pathways of *S aureus*. We indeed actually observed a lower SAIP incidence among *S aureus–*colonized patients in 1 region and/or a higher SAIP incidence in noncolonized patients in another region (Table 2).

In addition, the lowest risk for developing SAIP associated with *S aureus* carriage at admission was found in the in the region with the lowest genetic concordance between colonizing and infecting *S aureus* strains. This finding suggests that cross-transmission of *S aureus* contributed more to SAIP in this region than in regions with strong evidence for endogenous *S aureus* infection and with high heterogeneity in *S aureus* genotypes among patients with SAIP. This may have consequences for infection prevention. Targeted strategies interrupting progress from carriage to infection may be effective in settings where infections are predominantly of endogenous origin, whereas measures that reduce cross-transmission might be more effective in settings with indication of clonal transmission.

### Strengths and Limitations

The association between *S aureus* colonization and infection has been demonstrated before.^4,5,18,19^ The current study adds that there are regional differences in SAIP incidence, risk ratios between *S aureus*–colonized and noncolonized patients to develop SAIP, and medical practice associated with diagnostic culture frequencies. We consider the use of an objective definition for pneumonia, standardized laboratory screening methods, and sophisticated statistical analyses as strengths of the current study. However, despite the use of objective criteria, the diagnosis of pathogen-specific pneumonia depends on diagnostic practices, which varied from country to country. The definition of SAIP used in the current study is similar to definitions used in concurrent and upcoming intervention studies and was, as such, approved by the European Medicines Agency. Although the definition used included microbiological testing, it did not require quantitative measures, allowing pneumonia to be categorized as SAIP in case of low bacterial loads of *S aureus* or when other pathogens were also isolated. This may have caused misclassification and overestimation of the incidence of SAIP. On the basis of the current study, we, therefore, question the validity of the diagnostic criteria used for regulatory studies. With these definitions, trials investigating preventive or therapeutic measures may be biased to 0, or, in other words, would demonstrate unjustified absence of treatment effects.

Another study limitation is the incompleteness of outcome data in some countries. However, the number of patients for whom missing outcome data exceeded the predefined boundary was low (79 patients [4.1%]), and in 3 of these patients (3.8%), SAIP was observed despite missing data. This may have led to a slight underestimation of the SAIP incidence in patients with prolonged LOS. A sensitivity analysis in which these patients were excluded yielded similar results.

## Conclusions

In this cohort study, the overall incidence density of SAIP was 4.9 events per 1000 ICU days in patients undergoing mechanical ventilation at ICU admission (or shortly thereafter). Specifically, the SAIP incidence density was 11.7 events per 1000 ICU days for *S aureus*–colonized patients and 2.9 events per 1000 ICU days for noncolonized patients. *S aureus* colonization status was the only factor independently associated with SAIP occurrence, with a CSHR of 3.6 (95% CI, 2.2-6.0). Large regional differences in incidence rate and in CSHR for colonization status were observed.

## Notes

Supplement. **eTable 1. **Participating Countries and Total Included Subjects per Country **eTable 2. **Baseline Characteristics of Nonparticipants vs Complete Source Population, Stratified for SA Colonization Status **eTable 3. **SA ICU Pneumonia Categorization Based on Local and Study Culture Results **eTable 4. **Incidence of SA ICU Pneumonia (Unweighted) **eTable 5. **Incidence of SA ICU Pneumonia per Colonization Status per Region (Unweighted) **eTable 6. **Incidence of SA ICU Pneumonia per Colonization Status per Stratified for Sample Type (Weighted) **eTable 7. **Incidence of SA ICU Pneumonia per Colonization Status per Stratified for Sample Type (Unweighted) **eTable 8.** Average Number of Reported Cultures and Missing DPS (per Subject) **eTable 9. **Risk Factor Analysis (Univariable, Weighted) for Competing Events **eTable 10. **Risk Factor Analysis (Univariable and Multivariable, Unweighted) for SAIP **eTable 11. **Risk Factor Analysis (Univariable, Unweighted) for Competing Events **eFigure 1. **Cumulative Incidence Function (CIF) for SAIP and Competing Events (Unweighted) **eFigure 2. **CIF for SAIP per Colonization Status (Unweighted) **eFigure 3. **CIF for SAIP per Region (Weighted) **eFigure 4. **CIF for SAIP per Region (Unweighted) **eFigure 5. **CIF for SAIP per Colonization Status per Region (Weighted) **eFigure 6. **CIF for SAIP per Colonization Status per Region (Unweighted) **eFigure 7. **SAIP Incidence vs Average Number of Cultures per Subject per Day Over the 30 Sites **eAppendix. **Weighting Methods
