# Supplementary material for: Association of Staphylococcus aureus Colonization and Pneumonia in the Intensive Care Unit
Source: JAMA Netw Open. 2020 Sep 30;3(9):e2012741. doi: 10.1001/jamanetworkopen.2020.12741 (PMC7527877; doi:10.1001/jamanetworkopen.2020.12741)
Supplement: Supplement. — eTable 1. Participating Countries and Total Included Subjects per Country eTable 2. Baseline Characteristics of Nonparticipants vs Complete Source Population, Stratified for SA Colonization Status eTable 3. SA ICU Pneumonia Categorization Based on Local and Study Culture Results eTable 4. Incidence of SA ICU Pneumonia (Unweighted) eTable 5. Incidence of SA ICU Pneumonia per Colonization Status per Region (Unweighted) eTable 6. Incidence of SA ICU Pneumonia per Colonization Status per Stratified for Sample Type (Weighted) eTable 7. Incidence of SA ICU Pneumonia per Colonization Status per Stratified for Sample Type (Unweighted) eTable 8. Average Number of Reported Cultures and Missing DPS (per Subject) eTable 9. Risk Factor Analysis (Univariable, Weighted) for Competing Events eTable 10. Risk Factor Analysis (Univariable and Multivariable, Unweighted) for SAIP eTable 11. Risk Factor Analysis (Univariable, Unweighted) for Competing Events eFigure 1. Cumulative Incidence Function (CIF) for SAIP and Competing Events (Unweighted) eFigure 2. CIF for SAIP per Colonization Status (Unweighted) eFigure 3. CIF for SAIP per Region (Weighted) eFigure 4. CIF for SAIP per Region (Unweighted) eFigure 5. CIF for SAIP per Colonization Status per Region (Weighted) eFigure 6. CIF for SAIP per Colonization Status per Region (Unweighted) eFigure 7. SAIP Incidence vs Average Number of Cultures per Subject per Day Over the 30 Sites eAppendix. Weighting Methods [file jamanetwopen-e2012741-s001.pdf]

Supplemental Online Content

Paling FP, Hazard D, Bonten MJM, et al; ASPIRE-ICU Study Team. Association of *Staphylococcus aureus* colonization and pneumonia in the intensive care unit. *JAMA Netw Open*. 2020;3(9):e2012741. doi:10.1001/jamanetworkopen.2020.12741

eTable 1. Participating Countries and Total Included Subjects per Country

eTable 2. Baseline Characteristics of Nonparticipants vs Complete Source Population, Stratified for SA Colonization Status

eTable 3. SA ICU Pneumonia Categorization Based on Local and Study Culture Results

eTable 4. Incidence of SA ICU Pneumonia (Unweighted)

eTable 5. Incidence of SA ICU Pneumonia per Colonization Status per Region

(Unweighted)

eTable 6. Incidence of SA ICU Pneumonia per Colonization Status per Stratified for Sample Type (Weighted)

eTable 7. Incidence of SA ICU Pneumonia per Colonization Status per Stratified for Sample Type (Unweighted)

eTable 8. Average Number of Reported Cultures and Missing DPS (per Subject)

eTable 9. Risk Factor Analysis (Univariable, Weighted) for Competing Events

eTable 10. Risk Factor Analysis (Univariable and Multivariable, Unweighted) for SAIP

eTable 11. Risk Factor Analysis (Univariable, Unweighted) for Competing Events

eFigure 1. Cumulative Incidence Function (CIF) for SAIP and Competing Events (Unweighted)

eFigure 2. CIF for SAIP per Colonization Status (Unweighted)

eFigure 3. CIF for SAIP per Region (Weighted)

eFigure 4. CIF for SAIP per Region (Unweighted)

eFigure 5. CIF for SAIP per Colonization Status per Region (Weighted)

eFigure 6. CIF for SAIP per Colonization Status per Region (Unweighted)

© 2020 Paling FP et al. *JAMA Network Open.*

eFigure 7. SAIP Incidence vs Average Number of Cultures per Subject per Day Over the 30 Sites

eAppendix. Weighting Methods

This supplemental material has been provided by the authors to give readers additional information about their work.

© 2020 Paling FP et al. *JAMA Network Open*.

eTable 1. Participating Countries and Total Included Subjects per Country

Region	Country	Participants<br>This analysis	All
**North**	Estonia	145	151
	United Kingdom	106	106
**South**	Turkey	157	158
	Serbia	336	340
	Spain	329	338
**East**	Bulgaria	126	127
	Czech Republic	196	205
	Hungary	73	84
**West**	The Netherlands	328	344
	France	91	95
	Germany	46	49
**Total**		1,933	1,997

© 2020 Paling FP et al. *JAMA Network Open.*

eTable 2. Baseline Characteristics of Nonparticipants vs Complete Source Population, Stratified for SA Colonization Status

		Surveillance population				
		All*			Non-included	
as % (n) or mean (SD)		SA+ 24.8 (2,440)	SA- 69.5 (6,838)	SA ? 5.7 (563)	SA+ (1,433)	SA- (5,848)
**Age** n=2 missing		59.9 (17.0)	62.9 (15.1)	60.9 (16.4)	58.7 (17.0)	62.8 (15.1)
**Gender** n=1 missing	Male	66.2 (1,615)	62.7 (4,286)	62.5 (352)	65.8 (943)	62.6 (3,663)
	Female	33,8 (825)	37,3 (2,552)	37,3 (210)	34.2 (490)	37.4 (2,185)
**APACHE II score** n=4.346 missing		20.9 (9.1)	20.7 (8.8)	21.2 (9.2)	21.1 (9.6)	20.8 (8.9)
**Region**** n= 0 missing	North (1,790)	19.8 (483)	17.3 (1,182)	22.2 (125)	24.7 (354)	18.0 (1,054)
	South (2,755)	28.0 (682)	30,1 (2061)	1,8 (10)	18,1 (259)	28,2 (1648)
	East (1,192)	13.6 (332)	12.3 (838)	3.9 (22)	8.2 (118)	10.9 (636)
	West (4,106)	38.6 (943)	40.3 (2,757)	72.1 (406)	49.0 (702)	42.9 (2,510)
**Admission type** n= 3 missing	Medical	57.3 (1,399)	54.1 (3,699)	57.2 (322)	60.4 (866)	55.3 (3,232)
	Trauma	16.4 (401)	11.4 (777)	8.5 (48)	13.6 (195)	10.4 (606)
	Surgical cardiothoracic	6.3 (153)	9.6 (656)	13.7 (77)	7.2 (103)	10.0 (582)
	Surgical other	20.0 (487)	24.9 (1,706)	20.1 (113)	18.8 (269)	24.4 (1,428)
**Surgery** n=3 missing	Emergency	25.4 (620)	27.6 (1,889)	25.2 (142)	22.9 (328)	26.5 (1,549)
	Elective	8.5 (207)	12.2 (834)	11.5 (65)	9.1 (131)	12.6 (736)
**Neurotrauma** n=6 missing		10.2 (250)	6.2 (427)	3.9 (22)	9.0 (129)	5.8 (340)
**LOS (in days)** n=269 missing		12.1 (16.5)	11.6 (15.9)	7.9 (11.6)	10.8 (18.4)	11.2 (16.1)
**Death within ICU** n=266 missing		25.4 (609)	23.4 (1,550)	25.0 (138)	26.2 (365)	23.6 (1,329)

All characteristics were collected at ICU admission.
*incl. study cohort subjects.
** North= UK, Estonia, South= Spain, Turkey, Serbia, East= Czech, Bulgaria, Hungary, West=Netherlands, France, Germany. SD= standard deviation, SA= *S. aureus*. APACHE= Acute Physiology, Age, Chronic Health Evaluation.
Note: Subjects with SA pneumonia at ICU admission are included as participants in this overview.

© 2020 Paling FP et al. *JAMA Network Open*.

eTable 3. SA ICU Pneumonia Categorization Based on Local and Study Culture Results

**SA ICU pneumonia**		**Based on local cultures**		**Total**
		Yes	No	
**Based on study cultures**	Yes	63	57	120
	No<br>N/A	9<br>2	162<br>11	171
**Total**		74	230	304

N/A = no study sample available centrally.

© 2020 Paling FP et al. *JAMA Network Open.*

eTable 4. Incidence of SA ICU Pneumonia (Unweighted)

SA ICU pneumonia	**Days at risk**	**Risk N (%)**	**Rate N / 1000 days at risk**	**Time to SAIP (median, in days)**
**Colonization status**				
**- SA positive**	10,052	99 (10.4%)	9.8	5
**- SA negative**	11,409	32 (3.3%)	2.8	6
**Region**				
**- North**	2,602	22 (8.8%)	8.5	5
**- South**	10,864	52 (6.3%)	4.8	6.5
**- East**	3,905	24 (6.1%)	6.1	5.5
**- West**	4,090	33 (7.1%)	8.1	3
**Overall**	21,461	131 (6.8%)	6.1	5

SA= *S. aureus*, ICU= intensive care unit.

© 2020 Paling FP et al. *JAMA Network Open.*

eTable 5. Incidence of SA ICU Pneumonia per Colonization Status per Region (Unweighted)

SA ICU pneumonia per colonization status*	**Days at risk**	**Risk N (%)**	**Rate N / 1000 days at risk**	**Time to SAIP (median, in days)**
SA positive				
Region				
- North	1,218	17 (13.8%)	14.0	
- South	5,257	34 (8.3%)	6.5	
- East	1,771	19 (9.8%)	9.8	
- West	1,806	29 (13.0%)	13.0	
**Total (SA positive)**	10,052	99 (10.4%)	9.8	
SA negative				
Region				
- North	1,384	5 (3.9%)	3.6	
- South	5,607	18 (4.4%)	3.2	
- East	2,134	5 (2.5%)	2.3	
- West	2,284	4 (1.7%)	1.8	
**Total (SA negative)**	11,409	32 (3.3%)	2.8	

SA= *S. aureus*, ICU= intensive care unit. * measured at ICU admission

© 2020 Paling FP et al. *JAMA Network Open.*

eTable 6. Incidence of SA ICU Pneumonia per Colonization Status per Stratified for Sample Type (Weighted)

SA ICU pneumonia	**n**	**Days at risk**	**Risk N (%)**	**Rate N / 1000 days at risk**	**Time to SAIP (median, in days)**
**Nose**					
SA +	1,867	19,146	209 (11.2)	10.9	5
SA -	7,551	82,814	283 (3.7)	3.4	5
**ETA**					
SA +	822	7,400	170 (20.7)	23.0	4
SA -	6,409	74,342	240 (3.7)	3.2	6
**Throat**					
SA +	323	3,277	29 (9.0)	8.8	2
SA -	2399	21,774	72 (3.0)	3.3	15
**Sputum**					
SA +	34	193	4 (11.8)	20.7	2
SA -	280	2,194	4 (1.4)	1.8	3.5
**Overall**	9,425	101,977	495 (5.3)	4.9	4

SA= *S. aureus*, ICU= intensive care unit, SAIP=SA ICU pneumonia, ETA=endotracheal aspirate.

© 2020 Paling FP et al. *JAMA Network Open.*

eTable 7. Incidence of SA ICU Pneumonia per Colonization Status per Stratified for Sample Type (Unweighted)

SA ICU pneumonia	**n**	**Days at risk**	**Risk N (%)**	**Rate N / 1000 days at risk**	**Time to SAIP (median, in days)**
**Nose**					
SA +	809	8,706	80 (9.9)	9.2	5
SA -	1,122	12,750	50 (4.45)	3.9	6
**ETA**					
SA +	356	3,373	61 (16.9)	18.1	4
SA -	1,240	14,871	58 (4.7)	3.9	6
**Throat**					
SA +	127	1,345	10 (7.9)	7.4	3.5
SA -	362	3,343	11 (3.0)	3.3	5
**Sputum**					
SA +	16	72	1 (6.3)	13.9	2
SA -	52	411	2 (3.8)	4.9	3.5
**Overall**	1,933	21,461	131 (6.8)	6.1	5

SA= *S. aureus*, ICU= intensive care unit, SAIP=SA ICU pneumonia, ETA=endotracheal aspirate.

© 2020 Paling FP et al. *JAMA Network Open.*

eTable 8. Average Number of Reported Cultures and Missing DPS (per Subject)

	Local cultures / day (n)	Available sample centrally (%)	Missing DPS (n)	Missing DPS (%)
**North**	0.34	95.2	0.52	7.7
**South**	0.29	90.7	0.48	5.8
**East**	0.43	93.6	0.92	9.0
**West**	0.74	77.8	0.33	9.2
**All**	0.43	87.2	0.54	7.5

DPS= daily pneumonia score

© 2020 Paling FP et al. *JAMA Network Open.*

eTable 9. Risk Factor Analysis (Univariable, Weighted) for Competing Events

Risk factor	ICU death		ICU discharge	
	CSHR (95% CI)	p-value	CSHR (95% CI)	p-value
**SA colonization*** (non-colonized is reference category)	0.97 (0.77-1.21)	0.77	1.05 (0.93-1.18)	0.47
**Male gender** (female is reference category)	1.07 (0.80-1.42)	0.65	1.00 (0.86-1.16)	0.95
**Health care setting origin prior to ICU stay** (community is reference category)	1.07 (0.80-1.44)	0.64	0.82 (0.71-0.96)	**0.01**
**APACHE IV score*†**	1.01 (1.01-1.02)	**<0.001**	0.99 (0.99-1.00)	**<0.001**
**BMI†**	0.99 (0.97-1.01)	0.43	0.99 (0.98-1.00)	**0.12**
**Neurotrauma***	0.65 (0.44-0.97)	**0.04**	0.86 (0.72-1.03)	**0.11**
**Prior antibiotic use**	1.54 (1.14-2.01)	**0.005**	0.85 (0.71-1,01)	**0.07**
**Diabetes mellitus**	1.38 (0.99-1.93)	**0.06**	0.92 (0.78-1.09)	0.33
**Pneumonia***	1.33 (0.93-1.91)	**0.12**	0.76 (0.63-0.91)	**0.004**
**Active SA infection other than pneumonia ***	1.38 (0.70-2.72)	0.35	1.18 (0.80-1.74)	0.40
**Peptic ulcer prophylaxis#**	0.65 (0.38-1.11)	**0.11**	1.04 (0.82-1.33)	0.73
**Bed head elevation#**	0.80 (0.36-1.77)	0.59	0.40 (0.23-0.69)	**0.001**

Variables that univariably were associated with p-value <0.157 (**bold**) (for SAIP or competing events) were included in final multivariable model.
ICU= intensive care unit, CSHR= cause specific hazard ratio, CI= confidence interval, SA= *S. aureus*, APACHE= Acute Physiology, Age, Chronic Health Evaluation, BMI= body mass index.
*At ICU admission. †Per point increase. # During ICU stay.

© 2020 Paling FP et al. *JAMA Network Open.*

eTable 10. Risk Factor Analysis (Univariable and Multivariable, Unweighted) for SAIP

Risk factor	Univariable		Multivariable	
	CSHR (95% CI)	p-value	CSHR (95% CI)	p-value
**SA colonization*** (non-colonized is reference category)	3.44 (2.31-5.13)	**<0.001**	3.13 (2.1-4.7)	<0.001**
**Male gender** (female is reference category)	1.20 (0.83-1.75)	0.33	NI	
**Health care setting origin prior to ICU stay** (community is reference category)	0.58(0.40-0.84)	**0.004**	0.79 (0.52-1.19)	0.26
**APACHE IV score*†**	1.00 (1.00-1.01)	0.57	1.00 (1.00-1.01)	0.55
**BMI†**	0.98 (0.95-1.01)	0.16	0.98 (0.95-1.01)	0.21
**Neurotrauma***	1.63 (1.03-2.59)	**0.04**	1.22 (0.75-1.99)	0.42
**Prior antibiotic use**	0.41 (0.24-0.68)	**0.001**	0.56 (0.31-0.99)	0.047**
**Diabetes mellitus**	1.08 (0.70-1.69)	0.72	1.32 (0.83-2.09)	0.24
**Pneumonia***	0.61 (0.35-1.05)	**0.08**	0.79 (0.45-1.39)	0.41
**Active SA infection other than pneumonia ***	1.48 (0.60-3.67)	0.39	1.43 (0.57-3.60)	0.45
**Peptic ulcer prophylaxis[#]**	1.51 (0.78-2.90)	0.22	NI	
**Bed head elevation[#]**	0.74 (0.17-3.20)	0.69	0.96 (0.22-4.24)	0.96

Variables that were univariably associated with a p-value <0.157 (**bold**) (for SAIP or competing events) were included in final multivariable model assessing SAIP.
CSHR= cause specific hazard ratio, CI= confidence interval, SA= *S. aureus*, NI= not included, ICU= intensive care unit, APACHE= Acute Physiology, Age, Chronic Health Evaluation, BMI= body mass index.
*At ICU admission. †Per point increase. [#] During ICU stay. ** Significant in multivariable analysis.

© 2020 Paling FP et al. *JAMA Network Open.*

eTable 11. Risk Factor Analysis (Univariable, Unweighted) for Competing Events

Risk factor	ICU death		ICU discharge	
	CSHR (95% CI)	p-value	CSHR (95% CI)	p-value
**SA colonization*** (non-colonized is reference category)	1.01 (0.83-1.23)	0.92	1.04 (0.94-1.16)	0.46
**Male gender** (female is reference category)	0.95 (0.77-1.16)	0.59	1.03 (0.92-1.15)	0.66
**Health care setting origin prior to ICU stay** (community is reference category)	1.06 (0.86-1.30)	0.59	0.86 (0.77-0.97)	**0.009**
**APACHE IV score*†**	1.01 (1.01-1.02)	**<0.001**	0.99 (0.99-1.00)	**<0.001**
**BMI†**	0.99 (0.97-1.00)	**0.13**	0.99 (0.98-1.00)	**0.02**
**Neurotrauma***	0.81 (0.58-1.14)	0.23	0.85 (0.71-1.02)	**0.08**
**Prior antibiotic use**	1.39 (1.12-1.73)	**0.003**	0.83 (0.73-0.94)	**0.003**
**Diabetes mellitus**	1.23 (0.97-1.57)	**0.09**	0.92 (0.80-1.05)	0.22
**Pneumonia***	1.27 (0.99-1.62)	**0.06**	0.74 (0.63-0.85)	**<0.001**
**Active SA infection other than pneumonia ***	1.68 (0.98-2.88)	**0.06**	0.99 (0.70-1.38)	0.93
**Peptic ulcer prophylaxis#**	0.85 (0.54-1.34)	0.49	1.01 (0.82-1.24)	0.91
**Bed head elevation#**	0.53 (0.25-1.16)	**0.11**	0.72 (0.45-1.15)	0.17

Variables that univariably were associated with p-value <0.157 (**bold**) (for SAIP or competing events) were included in final multivariable model assessing SAIP).
*At ICU admission. †Per point increase. # During ICU stay.

© 2020 Paling FP et al. *JAMA Network Open.*

eFigure 1. Cumulative Incidence Function (CIF) for SAIP and Competing Events (Unweighted)

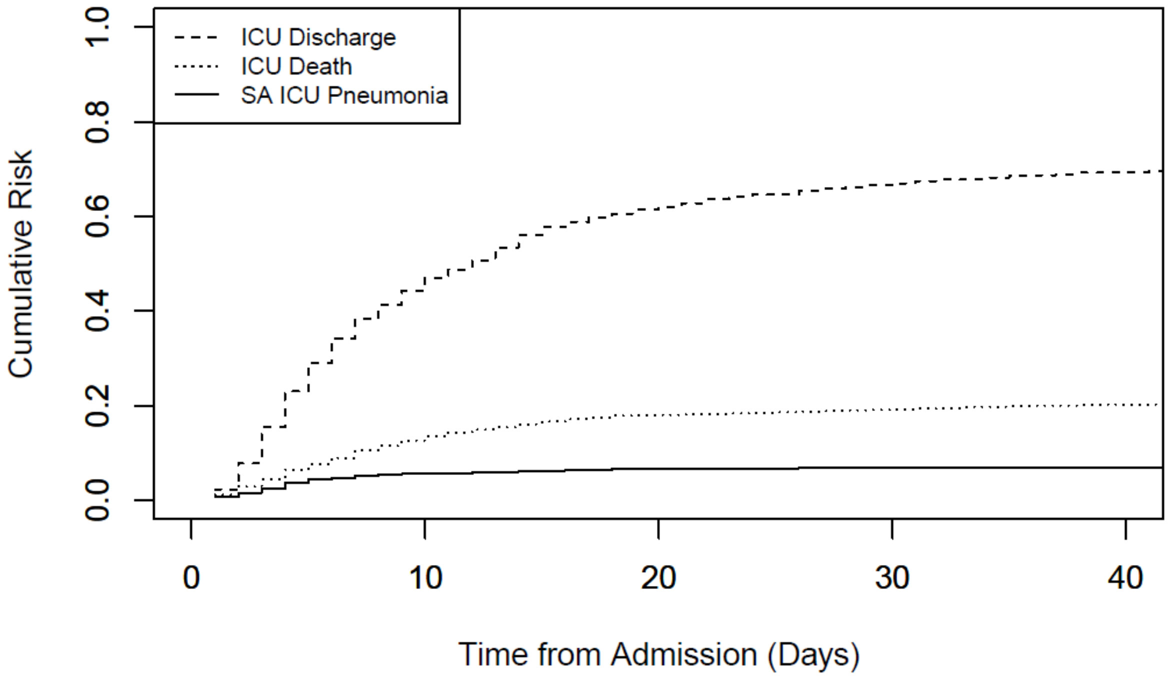

© 2020 Paling FP et al. *JAMA Network Open.*

eFigure 2. CIF for SAIP per Colonization Status (Unweighted)

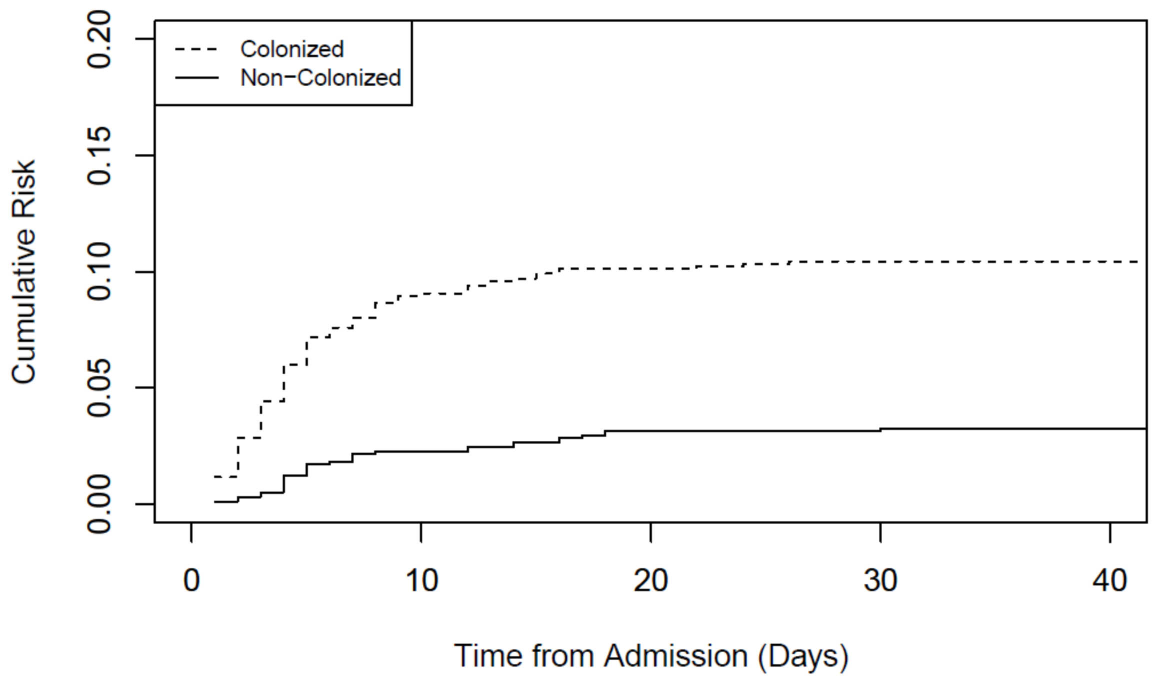

© 2020 Paling FP et al. *JAMA Network Open.*

eFigure 3. CIF for SAIP per Region (Weighted)

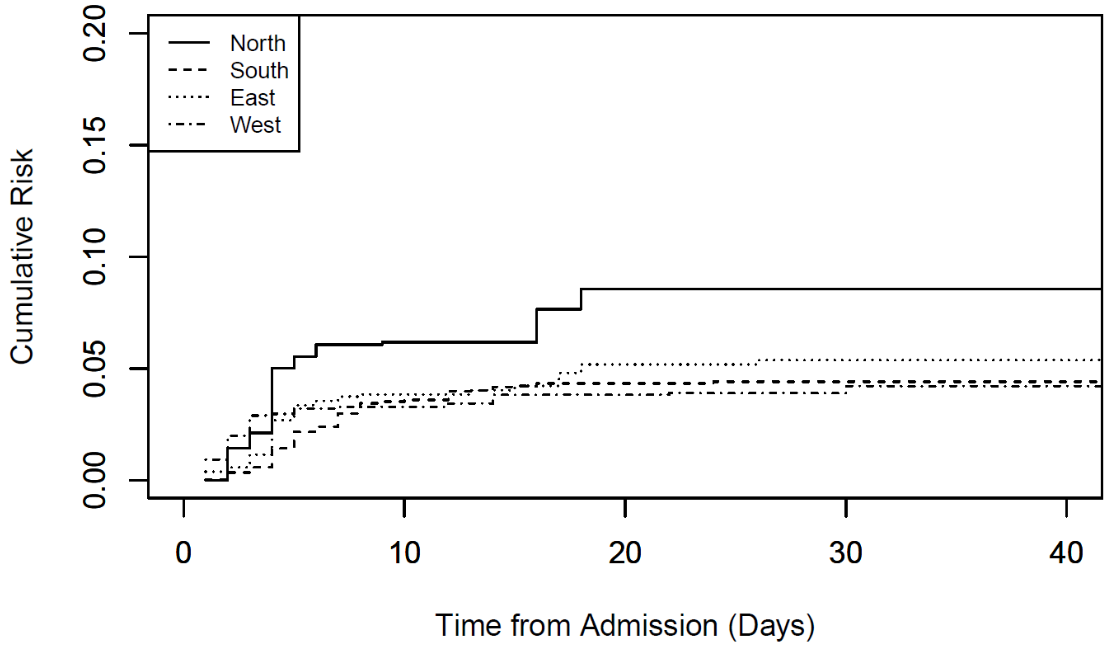

© 2020 Paling FP et al. *JAMA Network Open.*

eFigure 4. CIF for SAIP per Region (Unweighted)

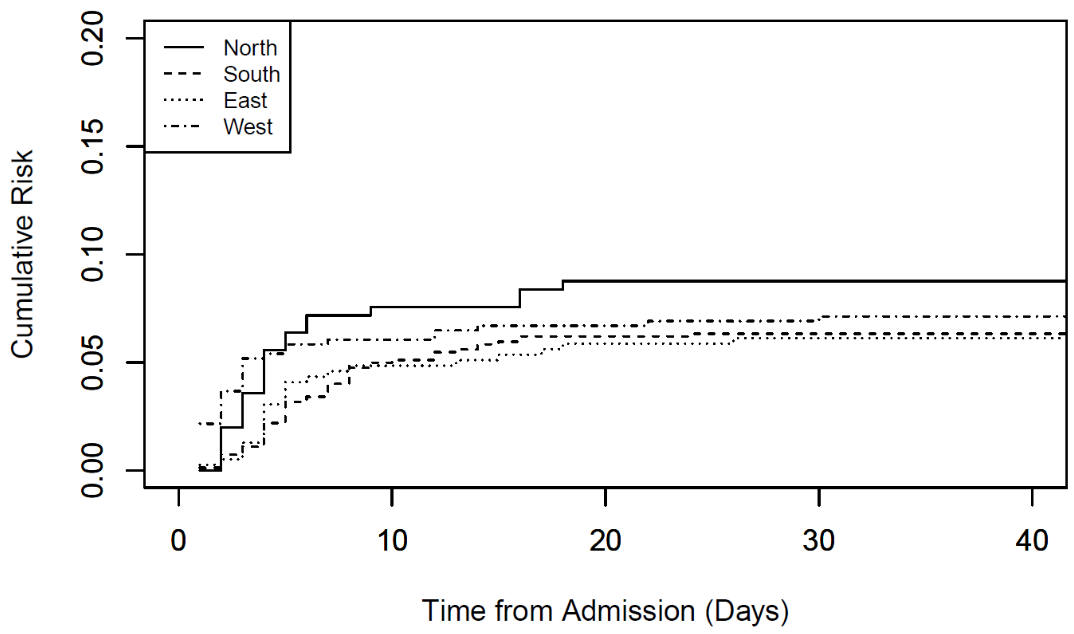

© 2020 Paling FP et al. *JAMA Network Open*.

eFigure 5. CIF for SAIP per Colonization Status per Region (Weighted).

a) North, b) South, c) East, d) West

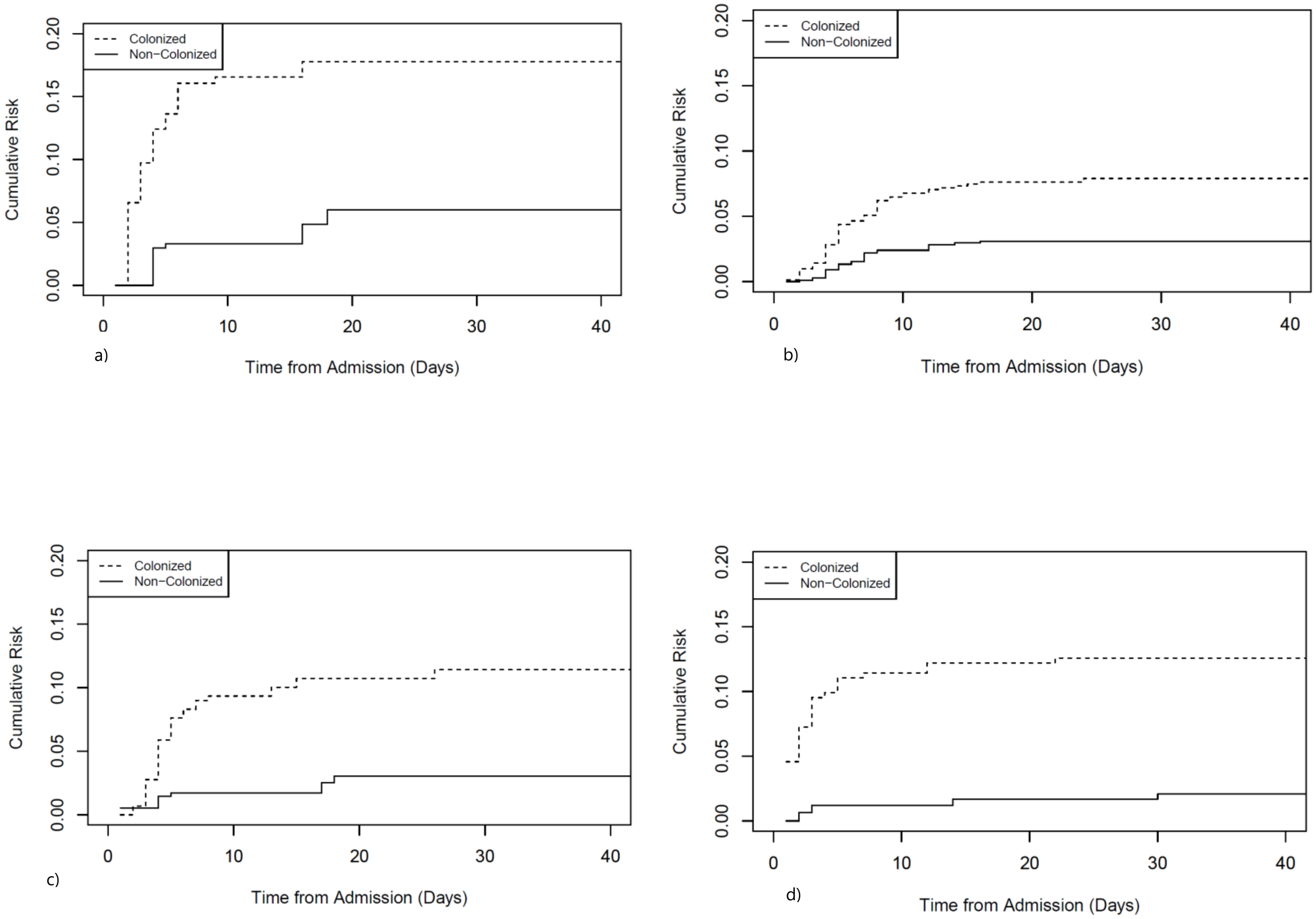

© 2020 Paling FP et al. *JAMA Network Open.*

eFigure 6. CIF for SAIP per Colonization Status per Region (Unweighted).
a) North, b) South, c) East, d) West

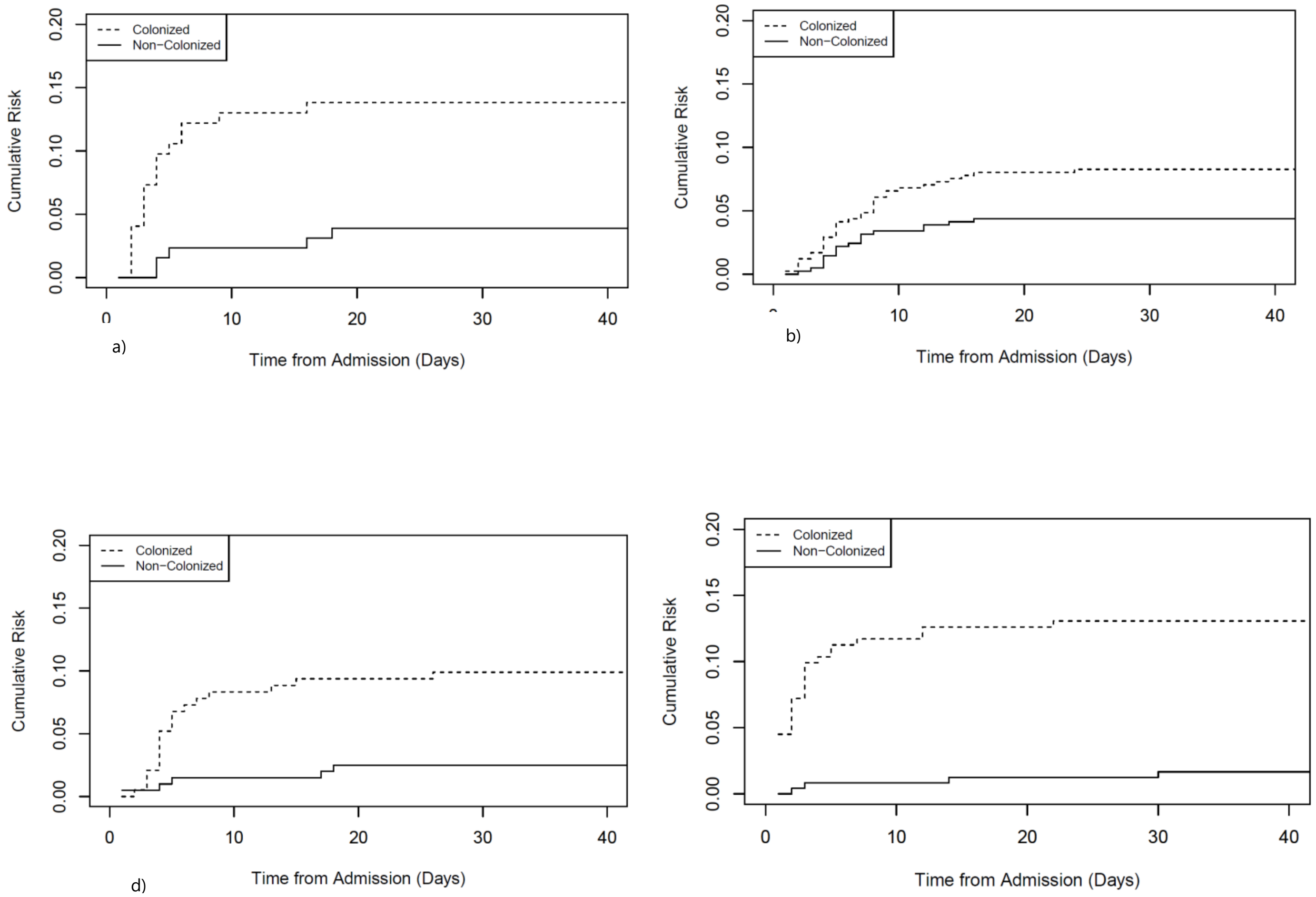

© 2020 Paling FP et al. *JAMA Network Open.*

eFigure 7. SAIP Incidence vs Average Number of Cultures per Subject per Day Over the 30 Sites.

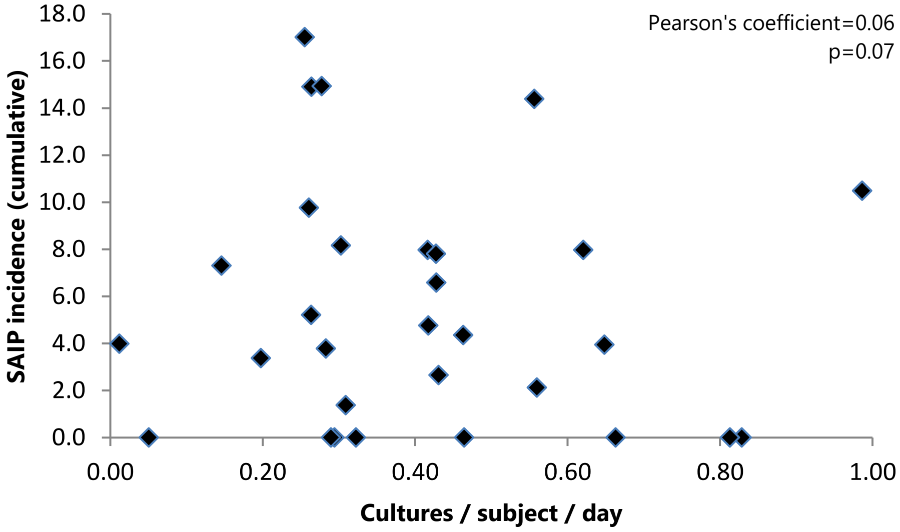

© 2020 Paling FP et al. *JAMA Network Open.*

eAppendix. Weighting Methods

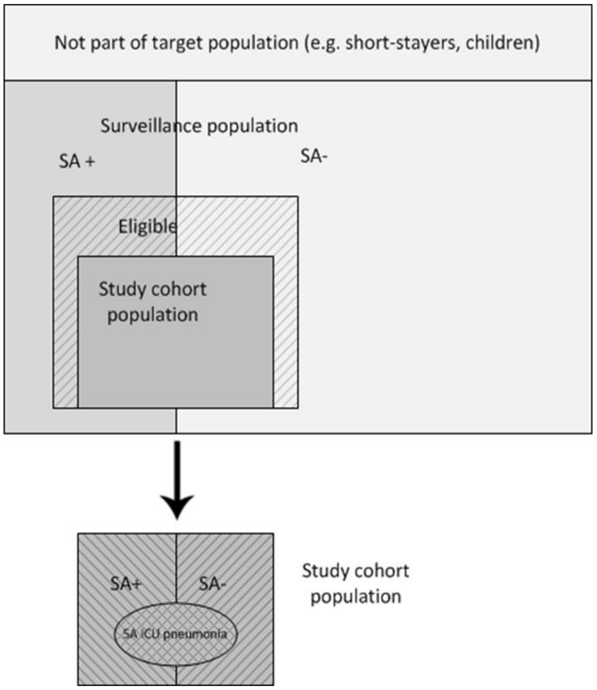

Due to the inclusion criteria of the ASPIRE-ICU study, SA colonized and non-colonized patients were included in the study cohort population at an approximately 1:1 ratio (see Figure 1). However, this ratio is approximately 1:3 in the overarching source population. The source population, in being a random sample of the general population, is the population we are interested in making inference on. Therefore, our aim was to recreate the source population by weighting the study cohort subjects with the inverse of the probability of their inclusion in the study cohort[1]. After conducting multiple imputation for missing values in the source population, we fitted a logistic regression model using predictor variables available for the source population, to estimate the inclusion probabilities. The predictor variables are listed in Table 1 below, as well as their characteristics in the source population, study cohort, source minus study cohort, and the weighted study population. There is good agreement between the source and weighted study population; most importantly in the colonization status proportions. The weights were subsequently used in the incidence and risk factor analyses in the accompanying manuscript.

© 2020 Paling FP et al. *JAMA Network Open.*

	level	Source	Study	Source-Study	Weighted
n		9841	1997	7844	9627
SA Colonization (%)	Yes	2440 (24.8)	1007 (50.4)	1433 (18.3)	2335 (24.3)
	No	6838 (69.5)	990 (49.6)	5848 (74.6)	7292 (75.7)
	Unknown	563 (5.7)	0 (0.0)	563 (7.2)	0 (0.0)
Country (%)	1	3153 (32.0)	344 (17.2)	2809 (35.8)	3026 (31.4)
	2	550 (5.6)	158 (7.9)	392 (5.0)	510 (5.3)
	3	443 (4.5)	127 (6.4)	316 (4.0)	420 (4.4)
	4	469 (4.8)	95 (4.8)	374 (4.8)	537 (5.6)
	5	721 (7.3)	340 (17.0)	381 (4.9)	710 (7.4)
	6	397 (4.0)	205 (10.3)	192 (2.4)	331 (3.4)
	7	1482 (15.1)	338 (16.9)	1144 (14.6)	1396 (14.5)
	8	352 (3.6)	84 (4.2)	268 (3.4)	339 (3.5)
	9	1302 (13.2)	106 (5.3)	1196 (15.2)	1397 (14.5)
	10	488 (5.0)	151 (7.6)	337 (4.3)	509 (5.3)
	11	484 (4.9)	49 (2.5)	435 (5.5)	452 (4.7)
AGE (mean (SD))		62.02 (15.69)	62.60 (15.92)	61.88 (15.64)	62.15 (15.12)
GENDER (%)	F	3587 (36.5)	702 (35.2)	2885 (36.8)	3507 (36.4)
	M	6253 (63.5)	1295 (64.8)	4958 (63.2)	6120 (63.6)
REASON (%)	MED	5420 (55.1)	1000 (50.1)	4420 (56.4)	5284 (54.9)
	SURG_CARD	886 (9.0)	124 (6.2)	762 (9.7)	896 (9.3)
	SURG_OTH	2306 (23.4)	496 (24.8)	1810 (23.1)	2289 (23.8)
	TRAUMA	1226 (12.5)	377 (18.9)	849 (10.8)	1158 (12.0)
TRAUMA_NEURO (%)	NO	9142 (92.9)	1789 (89.6)	7353 (93.7)	8946 (92.9)
	YES	699 (7.1)	208 (10.4)	491 (6.3)	681 (7.1)
ADM_YEAR (%)	2015	366 (3.7)	40 (2.0)	326 (4.2)	300 (3.1)
	2016	1976 (20.1)	316 (15.8)	1660 (21.2)	1947 (20.2)
	2017	4399 (44.7)	954 (47.8)	3445 (43.9)	4214 (43.8)
	2018	3100 (31.5)	687 (34.4)	2413 (30.8)	3166 (32.9)
Surgery (%)	ELEC	1106 (11.2)	174 (8.7)	932 (11.9)	1169 (12.1)
	EMERG	2651 (26.9)	632 (31.6)	2019 (25.7)	2593 (26.9)
	NO SURG	6084 (61.8)	1191 (59.6)	4893 (62.4)	5865 (60.9)
APACHE_II_SCORE (mean (SD))		20.76 (8.90)	20.53 (8.58)	20.85 (9.03)	21.12 (8.87)

© 2020 Paling FP et al. *JAMA Network Open.*
